# Effect of tibial tray design on cement morphology in total knee arthroplasty

**DOI:** 10.1186/s13018-014-0123-2

**Published:** 2014-11-29

**Authors:** Ulf J Schlegel, Klaus Püschel, Michael M Morlock, Katrin Nagel

**Affiliations:** Center for Orthopedics and Trauma Surgery, Heidelberg University Hospital, Schlierbacher Landstr 200a, 69118 Heidelberg, Germany; Institute of Legal Medicine, University of Hamburg-Eppendorf, Butenfeld 34, 22529 Hamburg, Germany; Biomechanics Section, TUHH Hamburg University of Technology, Denickestr 15, 21073 Hamburg, Germany

**Keywords:** Total knee arthroplasty, Cementing technique, Mobile bearing, Cement pockets, Bone cement penetration

## Abstract

**Background:**

Improvements to enforce primary fixation in cemented total knee arthroplasty have been suggested to be a key issue for long-term survival. In this context, it has been questioned whether specific implant design features influence bone cement morphology and hence primary interface strength. The purpose of this study was to investigate *in vitro* the influence of cement pockets on the tibial tray on cement penetration in the tibia.

**Methods:**

Eight paired cadaveric, human tibiae were available for investigation. One side of a pair was implanted with a fixed bearing tibial tray (FB) featuring cement pockets on the undersurface, while in the other side, a mobile bearing platform (MB) without cement pockets was used. Specimens underwent computed tomography analysis of the cement morphology as well as BMD assessment.

**Results:**

While bone cement layer between implant and bone surface was thicker in the FB group (*p* = 0.032), bone cement penetration was not influenced by implant design (*p* = 0.529).

**Conclusions:**

The present study suggests that cement pockets do not alter or enforce bone cement penetration under the tibial tray in an *in vitro* scenario.

## Background

Cemented total knee arthroplasty (TKA) is widely accepted as the most effective treatment for end-stage osteoarthritis of the knee. Although favorable clinical results have been reported, aseptic loosening of the tibial component remains troublesome [1-3]. As the need for revision surgery in total knee arthroplasty increases steadily, there is growing interest to improve initial fixation [4]. Aseptic loosening can be attributed to continuous micromotion at the implant-cement or bone-cement interface [5]. There is increasing evidence that the interface strength of the cemented fixation at the time of surgery is a major factor determining the long-term performance of the implant [6,7]. However, there is little information regarding bone cement penetration characteristics with newer tray designs for alternative bearing philosophies such as mobile bearing platforms. There has been a higher prevalence of tibial failure with early component designs, which highlights the role of the implant itself in the loosening process [8]. Implants featuring a peripheral lip on the undersurface have been suggested to enforce cement penetration, but the presence is depending on the manufacturer and the design [9]. Another biomechanical study suggested likewise that surface preparation and type of metal substrate may influence the bonding of the tibial component to the cement [10]. Thus, it can be questioned if cement mantle morphology differs among tibial trays of different designs. Design features on the undersurface of the component could influence bone cement penetration and likewise morphology in the trabecular bone, which could alter initial fixation strength. Hence, the aim of this study was to evaluate and compare bone cement penetration patterns in the tibial cancellous bone between different tibial tray designs by computed tomography (CT) scans and 3D imaging.

## Methods

**Figure 1:**
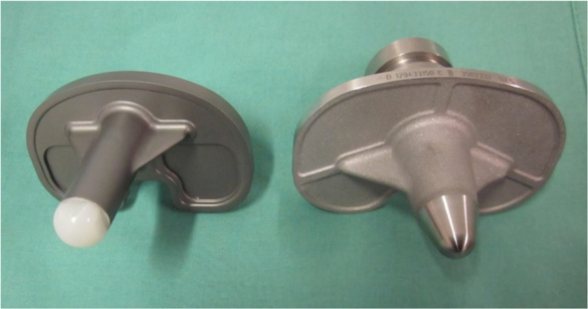
**Undersurface of FB with posterior cement pockets (left) and MB (right).**

**Figure 2:**
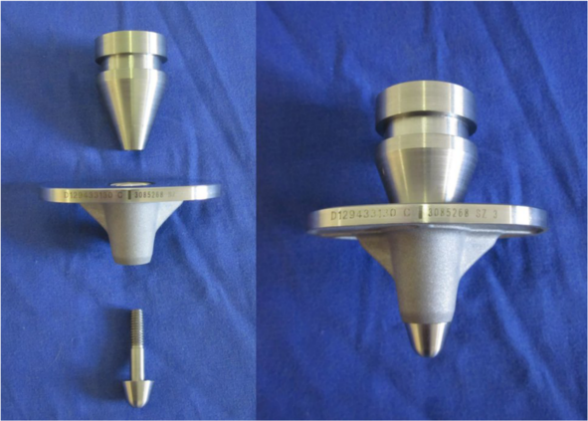
**Modification for testing of MB trays: disassembled (left) and assembled (right).**

The study was conducted using eight paired proximal, cadaveric tibiae. The specimens were conserved in a freezer at −30°C and thawed overnight before experiments were carried out at room temperature (22°C). The age of the donors was 82 years at median ranging from 71 to 87. All tibiae underwent CT analysis for determination of BMD and to exclude specimens with osseous aberrations (Brilliance 40-channel, Philips Medical Systems, Haifa, Israel). BMD was assessed relating Hounsfield units in a rectangular volume of 4,000 cm^3^ in the tibial head to a calibration normal (Avizo 5.0, VSG, Burlington, MA, USA). Two different tibial components were investigated in the study: P.F.C. Sigma Ti Fixed Bearing (FB) and P.F.C. Sigma MBT Keeled (mobile bearing = MB). The FB trays are made of titanium, while the latter consists of a cobalt chromium alloy (DePuy Orthopaedics, Warsaw, IN, USA). Both trays are designed for cemented fixation. While the P.F.C. FB provides a peripheral lip with posterior cement pockets with undercuts on the undersurface, the P.F.C. MB provides a smaller peripheral and central lip without specific pockets and undercuts (Figure 1). To facilitate implantation and handling, a screw thread was cut into the FB trays. This was not possible in the MB trays due to the cone-shaped opening for the inlay. Therefore, the tip of the stem was cut off and replaced by a tip featuring an inside screw (Figure 2). The specimens were then prepared for implantation of the tibial trays (size 3) following the manufacturer’s guidelines using a 0° slope cutting block and corresponding punches (DePuy Orthopaedics, Warsaw, IN, USA). All surgeries were carried out by the first author. In the final preparation step, all bones were cleaned using a syringe lavage and 1,800 ml saline solution. Pulsed lavage was not used as pilot measurements showed that the effect of pulsed lavage could overlay any other cementing effect. After careful drying, bone cement was vacuum mixed (60 s) and hand pressurized on the tibial surfaces after a waiting period of 120 s (Smartset HV 40 g, DePuy Orthopaedics, Warsaw, IN, USA). The stem of the implant was left cementless (surface cementing technique). The components were impacted by ten mallet blows; a steel lid was applied after impaction to apply constant axial loading of 50 N until the cement was cured. The specimens were then again evaluated by CT scans for analysis of the cement morphology in detail. The median cement layer thickness extending from the undersurface of the implant to the bony surface was determined. Accordingly, the median bone cement penetration reaching from the osseous surface into the cancellous bone was analyzed. This was done using a previously established method with a 3D imaging software and a numerical computing package (Avizo 5.0, VSG, Burlington, MA, USA and MATLAB, The MathWorks Inc., Natick, MA, USA), as outlined in detail elsewhere [11]. Statistical analysis comparing cement layer thickness and penetration depth was performed by a paired *t*-test provided that the distribution of the data is parametric (GraphPad Software, Inc., La Jolla, CA, USA). BMD as a structural parameter was controlled for by the paired design and also compared between designs using one-way ANOVA. A type I error probability of 5% was used for all tests. Continuous data were described by mean and standard deviation or—where applicable—by median and range.

## Results

**Figure 3:**
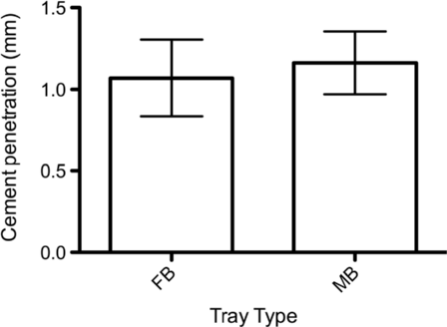
**Bone cement penetration depths for the four tibial pairs (mean and standard deviation).**

**Figure 4:**
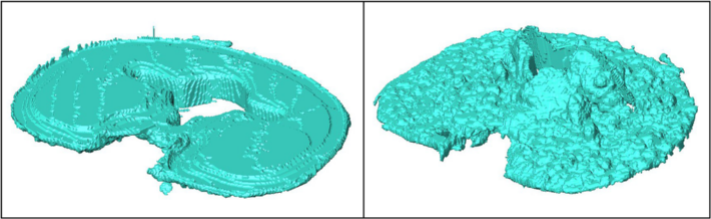
**Examples of 3D reconstructions of P.F.C. Sigma® Ti Fixed Bearing bone cement mantles.** Left: top view, right: undersurface.

**Figure 5:**
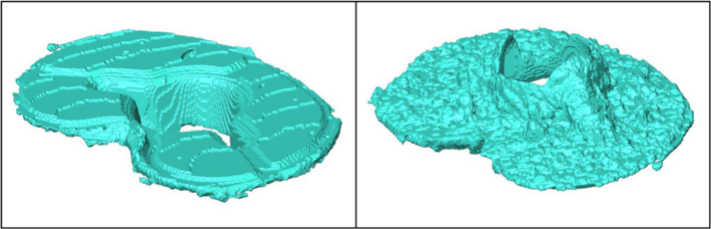
**Examples of 3D reconstructions of P.F.C. Sigma® MBT Keeled bone cement mantles.** Left: top view, right: undersurface.

BMD was similar for the two tray design groups (FB: 64 mg/cm^3^, SD = 45 mg/cm^3^; MB: 63 mg/cm^3^, SD = 37 mg/cm^2^; *p* = 0.967; Table 1). The mean bone cement penetration was also similar: 1.07 mm (SD = 0.23 mm) for the FB and 1.16 mm (SD = 0.19 mm) for the MB group (*p* = 0.529; Figure 3). Cement layer thickness between the undersurface of the implant and the osseous surface was higher for the FB (2.32 mm, SD = 0.22 mm) than for the MB (1.47 mm, SD = 0.27 mm, *p* = 0.032). Examples for the cement mantle reconstructions generated from the CT data are shown in Figures 4 and 5.

**Table 1:** **Specimen characteristics and results (F = female, M = male)**

**Specimen**	**Sex**	**Age**	**Tray type**	**BMD (mg/cm** ^**3**^ **)**	**Cement layer (mm)**	**Penetration (mm)**
1R	M	71	MB	16	1.14	1.40
1 L			FB	13	2.64	0.96
2R	F	81	FB	55	2.25	1.42
2 L			MB	62	1.65	1.23
3R	F	87	MB	65	1.37	1.05
3 L			FB	66	2.12	0.97
4R	M	83	FB	122	2.26	0.93
4 L			MB	106	1.73	0.97

## Discussion

Differences in tibial component design and surgical technique influence the long-term performance of the implant regarding early aseptic loosening in TKA [12]; cementing techniques and choice of implant design still are controversial [13,14]. It has been shown that a peripheral lip improves bone cement penetration into the osseous surface [9]. The present study investigated *in vitro* whether cement pockets on the undersurface could likewise influence bone cement penetration characteristics. A cement pocket could possibly increase penetration by entrapment of bone cement in the undercutting area and directing it into the cancellous bone surface. However, the effect could not be observed in this *in vitro* scenario. Overall cement penetration was similar in the FB and MB group. The results from Vertullo and Davey on 177 consecutive TKA showed twice as deep cement penetration in lipped baseplates compared to unlipped ones [9]. However, the data was derived from radiographs, which is questionable as the implants and the keels obscure some of the cemented area. In our experience, accuracy of discrimination between cement under the baseplate and real penetration is difficult and error-prone under those circumstances. Furthermore, the actual cement penetration depth into the bone could not be visualized by the cited study setup due to the interference of cement with flanges and stem and only the lateral part of the tray was studied. In our observation, the overall penetration depths were similar in both groups, as we could not observe a significant increase in cement penetration in the FB group. On the other hand, a cement pocket may enforce rotational stability. However, this issue was not investigated in the present study. Penetration depths in our setting ranged from 0.93 to 1.42 mm and match perfectly with the data retrieved in a preceding investigation [11]. A mean cement penetration of 3 to 4 mm has been suggested to be the optimum for implant fixation [15-17]. Comparing the values to our data indicates that former studies could not differentiate between cement layer under the tray and actual penetration. Hence, cement penetration has probably been reported as a combination of both components, which might explain the observed differences. Further studies on bone cement penetration should focus on the “real” penetration into cancellous bone, as presented here. The thickness of the cement layer extending from the undersurface of the implant to the osseous surface was higher in the FB group (mean 2.32 mm) compared to the MB group (mean 1.47 mm). This effect is most likely related to the cement pockets of the FB implant and suggests that cement layer thickness is mainly influenced by implant design. Whether a thicker cement mantle is beneficial or detrimental is unclear and needs further research.

The small sample size is a limitation of the study. However, since the effects were similarly observed for each pair, the small sample size should not have biased the results. The transfer of *in vitro* testing to *in vivo* conditions remains limited, which has to be kept in mind when interpreting the presented results.

## Conclusions

In summary, the present study suggests that cement pockets with undercuts do not alter or enforce bone cement penetration under a tibial tray in an *in vitro* scenario.
